# Supplementary material for: A snapshot of biodiversity protection in Antarctica
Source: Nat Commun. 2019 Feb 26;10:946. doi: 10.1038/s41467-019-08915-6 (PMC6391489; doi:10.1038/s41467-019-08915-6)
Supplement: Supplementary file 1 — Supplementary Information [file 41467_2019_8915_MOESM1_ESM.pdf]

Supplementary Information

A snapshot of biodiversity protection in Antarctica

Hannah Wauchope[1], Justine D. Shaw[2], Aleks Terauds[3, *]

[1] Department of Zoology, University of Cambridge, The David Attenborough Building, Pembroke Street, Cambridge, CB2 3QZ, United Kingdom

[2] School of Biological Sciences, The University of Queensland, St Lucia, Queensland, 4067, Australia

[3] Antarctic Conservation and Management Program, Australian Antarctic Division, Kingston, Tasmania, 7050, Australia

[*] Corresponding author

Supplementary Tables and Figures

Supplementary Table 1: The nine protocol designation categories

Supplementary Table 2: Details on each ASPA

Supplementary Figure 1: Variety of statistics shown by Antarctic Conservation Biogeographic region (ACBR).

Supplementary Figure 2: Species richness at a continental and regional scales.

Supplementary Figure 3: Top ten taxa by area (km^2) protected
.
Supplementary Figure 4: ASPA coverage by primary designation criteria.

Supplementary Table 1. The nine reasons for designating an ASPA according to the Protocol on Environmental Protection to the Antarctic Treaty. Taken from https://www.ats.aq/devPH/apa/ProtectedAreas_Report.aspx (last accessed 6th August, 2018).

Identifier	Details of Designation
**A**	Areas kept inviolate from human interference so that future comparisons may be possible with localities that have been affected by human activities.
**B**	Representative examples of major terrestrial, including glacial and aquatic, ecosystems and marine ecosystems.
**C**	Areas with important or unusual assemblages of species, including major colonies of breeding native birds or mammals.
**D**	The type locality or only known habitat of any species.
**E**	Areas of particular interest to ongoing or planned scientific research.
**F**	Examples of outstanding geological, glaciological or geomorphological features.
**G**	Areas of outstanding aesthetic and wilderness value.
**H**	Sites or monuments of recognised historic value.
**I**	Areas as may be appropriate to protect the values set out in Article 3 paragraph1 of Annex V.

Supplementary Table 2. List of Antarctic Specially Protected Areas (ASPAs), with area km^2, primary protocol designation (see Supplementary Table 1), whether the ASPA was primarily designated (at least in part) to protect biodiversity and whether it is a marine ASPA (1 is yes, 0 is no), and finally the Antarctic Conservation Biogeographic Region (ACBR, see Supplementary Figure 1) the ASPA falls within.

ASPA Number	Area (km^2)	Primary Protocol designation	Biodiversity designated	Marine	ACBR ID
**101**	0.27	C	1	0	16
**102**	0.85	C	1	0	16
**103**	2.51	C	1	0	7
**104**	0.42	B	1	0	NA
**105**	13.76	C	1	0	9
**106**	0.53	C	1	0	8
**107**	4.77	C	1	0	3
**108**	0.18	C	1	0	3
**109**	1.35	B	1	0	2
**110**	0.13	B	1	0	2
**111**	5.74	C	1	0	2
**112**	0.71	B	1	0	3
**113**	0.38	C	1	0	3
**114**		De-designated			
**115**	1.65	B	1	0	3
**116**	0.33	C	1	0	9
**117**	1.26	C	1	0	3
**118**		De-designated			
**119**	53.68	C	1	0	10
**120**	0.38	B	1	0	13
**121**	0.64	C	1	0	9
**122**	0.71	I	0	0	9
**123**	410.80	G	1	0	9
**124**	67.65	C	1	0	9
**125**	2.45	F	0	0	3
**126**	92.96	C	1	0	3
**127**	6.07	E	1	0	7
**128**	18.38	C	1	0	3
**129**	0.04	A	1	0	3
**130**		De-designated			
**131**	1.45	C	1	0	9
**132**	1.79	B	1	0	3
**133**	46.36	C	1	0	3
**134**	18.19	C	1	0	3
**135**	0.29	C	1	0	7
**136**	9.68	C	1	0	7

**137**	136.96	C	1	0	9
**138**	0.76	C	1	0	9
**139**	0.62	C	1	0	3
**140**	2.92	E	1	0	3
**141**	5.22	B	1	0	5
**142**	7.61	C	1	0	6
**143**	20.75	B	1	0	7
**144**	0.72	E	1	1	NA
**145**	2.33	E	1	1	NA
**146**	1.00	E	1	1	NA
**147**	102.08	F	0	0	4
**148**	0.21	F	0	0	1
**149**	11.22	E	1	0	3
**150**	1.26	C	1	0	3
**151**	1.54	C	1	0	3
**152**	960.63	E	1	1	NA
**153**	636.57	C	1	1	NA
**154**	1.92	C	1	0	9
**155**	0.05	H	0	0	9
**156**	13.97	I	0	0	9
**157**	0.04	H	0	0	9
**158**	0.0001	H	0	0	9
**159**	0.02	H	0	0	8
**160**	0.62	C	1	0	7
**161**	29.41	E	1	1	NA
**162**	1.05	H	0	0	13
**163**	4.57	E	1	0	6
**164**	10.29	C	1	0	16
**165**	5.51	A	1	0	8
**166**	0.17	H	0	0	13
**167**	2.21	C	1	0	7
**168**	101.70	F	0	0	16
**169**	17.14	C	1	0	7
**170**	106.40	C	1	0	4
**171**	1.04	C	1	0	3
**172**	421.72*	F	0	0	9
**173**	279.99	C	1	0	8
**174**	21.31	F	0	0	7
**175**	0.26	C	1	0	8,9

*Note that ASPA 172s area consists largely of a subglacial boundary, which is thought to contain a subglacial marine salt deposit and brine reservoir. Though microbial life may exist within this area, there is no certainty regarding what exists in the subglacial area, or where, and for this reason we have chosen to exclude this from our analysis. In our analyses the

subsurface area of ASPA 172 is considered and is $0.11km^2$. Relevant text from the ASPA 172 Management Plan (available at https://ats.aq/devPH/apa/ep_protected_detail.aspx?type=2&id=165&lang=e, last accessed 3rd Nov, 2018): "Available evidence suggests the source of the discharge is a subglacial marine salt deposit and brine reservoir located beneath the Taylor Glacier… The Blood Falls outflow contains a unique microbial community of apparently marine origin. The microbes may survive in the subglacial environment for millions of years without external carbon input. On account of its high iron and salt content, the microbial ecosystem at Blood Falls is an important site for exobiological studies and may provide an analogue for the conditions found beneath the polar ice caps on Mars. It is therefore important to ensure that the Blood Falls microbial community, the brine reservoir and associated subglacial hydrological system are protected."

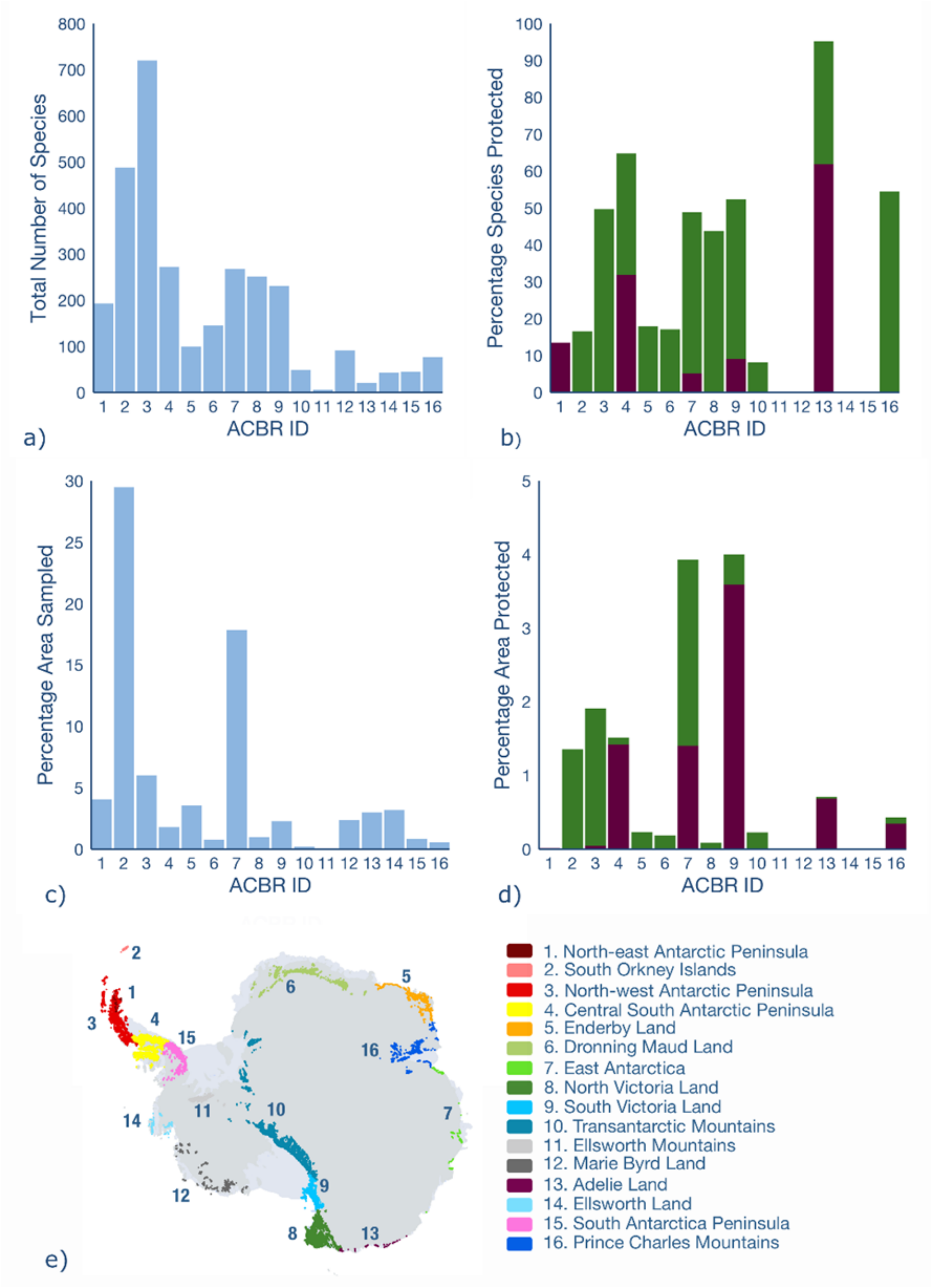

Supplementary Figure 1. Variety of statistics shown by Antarctic Conservation Biogeographic region (ACBR). (a) Total number of species occurring in each ACBR; (b) percentage of species occurring in at least one ASPA, split by percentage of species occurring in at least one biodiversity ASPA (green) and percentage of species not occurring in any biodiversity ASPA (purple); (c) percentage area sampled, calculated by number of 1 km grid cells of ACBR that have at least one species record; (d) percentage area of ACBR protected by ASPAs, split by biodiversity ASPAs (green) and non-biodiversity ASPAs (purple); and (e) names and location of each ACBR. Source data and code used to produce this figure are provided in Supplementary Datasets 2 and 3 and Supplementary Code 1. The Antarctic map (1e) uses the coastline layer from the SCAR Antarctic Digital Database (https://www.add.scar.org/) and the ACBR layer from the Australian Antarctic Data Centre[38].

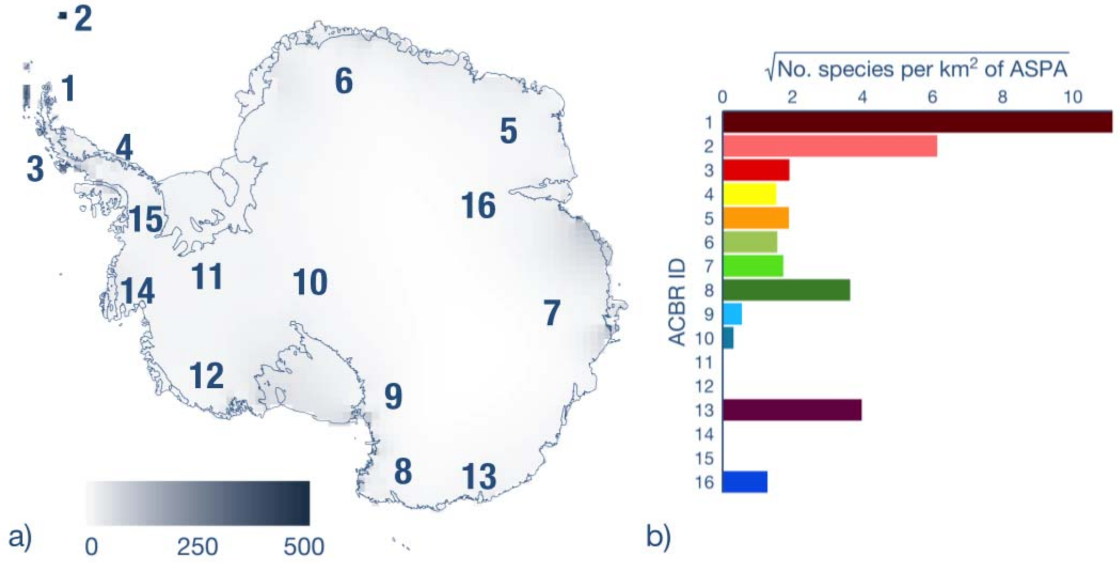

Supplementary Figure 2. Species richness at a continental and regional scales. (a) Heat map showing species richness on the Antarctic continent, and location the 16 Antarctic Conservation Biogeographic Regions. Figure produced by quantifying richness within 65 km^2 grid-cells (using the Antarctic dataset) and using linear kriging to interpolate this value to surrounding grid-cells (to aid in visualization). This map should therefore be viewed as an approximate representation of species richness around the continent. (b) Bar plot showing number of species protected (square root transformed) per km^2 of ASPA in each ACBR. Source data and code used to produce this figure are provided in Supplementary Datasets 2,3 and 4 and Supplementary Code 1. The Antarctic map (2a) uses the coastline layer from the SCAR Antarctic Digital Database (https://www.add.scar.org/).

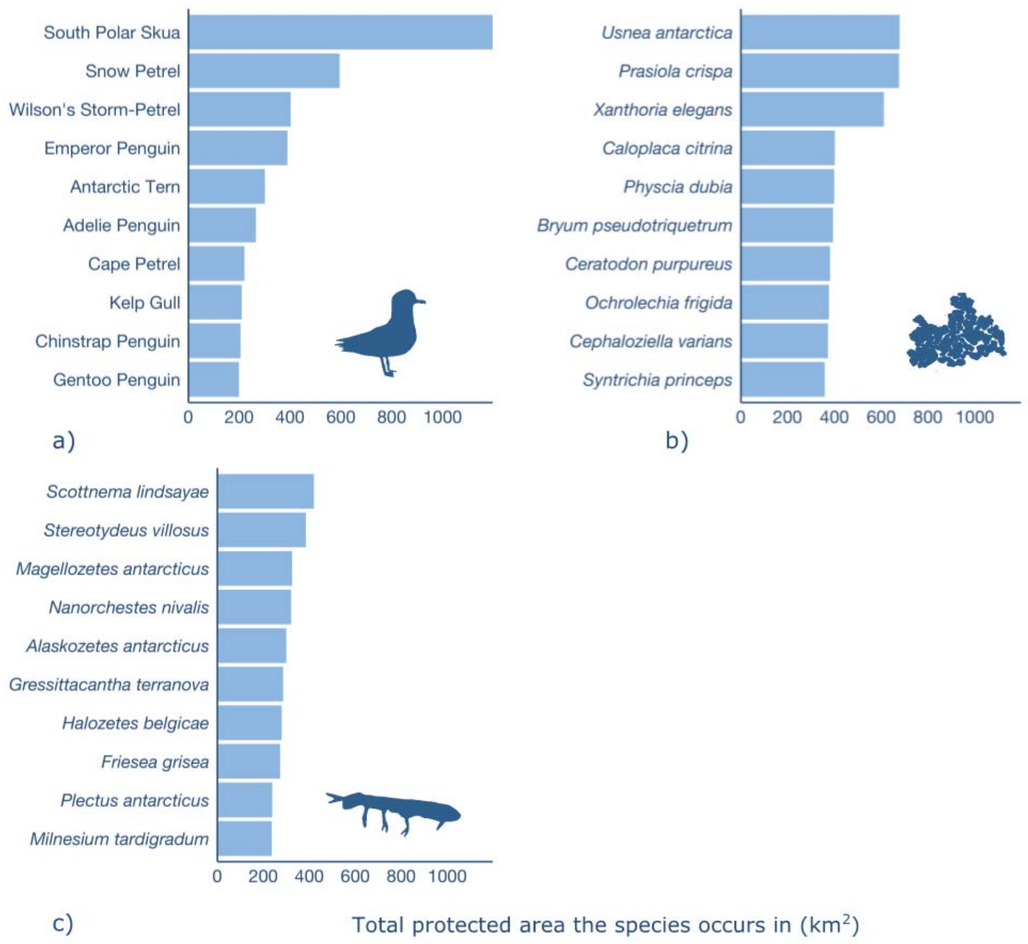

Supplementary Figure 3. Top ten taxa by area (km^2) protected*. (a) birds; (b) plants/lichens; and (c) invertebrates. Source data and code used to produce this figure are provided in Supplementary Datasets 2 and 5 and Supplementary Code 1.

*Note that point records from within ASPAs do not generally give an indication of the distribution of the species within the ASPA, and so these statistics should be viewed as the maximum possible range protected for the species, but the actual value may be much lower. These results are also biased by a small number of very large ASPAs, including 126, 147 and 170, in which many of these species occur. Despite these biases, there is reasonably high convergence between the best protected species by area (this figure), and best protected by number of ASPAs (Figure 1d), with 8/10 chordates, 6/10 plant/lichen and 5/10 invertebrates being the same between the two.

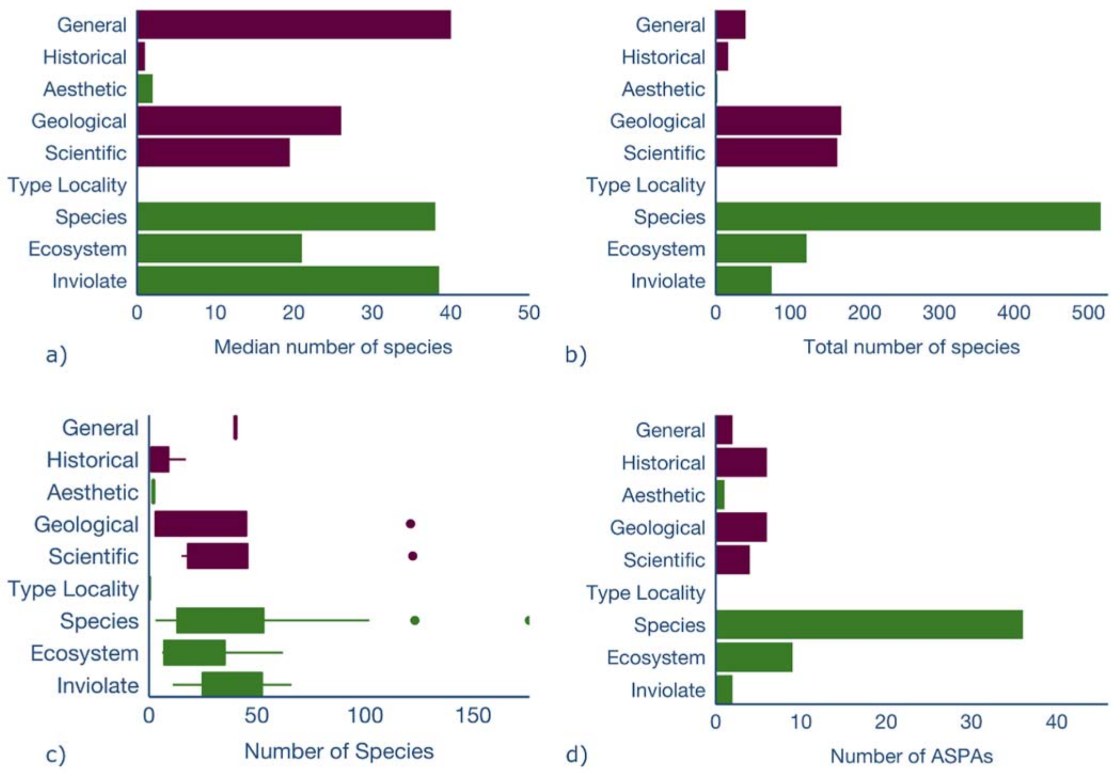

Supplementary Figure 4. ASPA coverage by primary designation criteria. In all cases green refers to criteria that include biodiversity protection, and purple to criteria that do not. (a) median number of species occurring in ASPAs of each designation, as shown in main infographic (b) total number of species occurring in ASPAs of each designation; (c) boxplot of number of species occurring in ASPAs of each designation; and (d) number of ASPAs of each designation. Source data and code used to produce this figure are provided in Supplementary Dataset 2 and Supplementary Code 1.
