# Supplementary material for: A snapshot of biodiversity protection in Antarctica
Source: Nat Commun. 2019 Feb 26;10:946. doi: 10.1038/s41467-019-08915-6 (PMC6391489; doi:10.1038/s41467-019-08915-6)
Supplement: Supplementary file 3 — Description of Additional Supplementary Files [file 41467_2019_8915_MOESM3_ESM.pdf]

Description of Additional Supplementary Files

File Name: Supplementary Data 1

Description: A detailed description of all the Supplementary Datasets

File Name: Supplementary Data 2

Description: Records of which species occur in each of the 16 Antarctic Conservation Biogeographic Regions (ACBRs,) and which species occur in each of the 72 Antarctic Specially Protected Areas (ASPAs)

File Name: Supplementary Data 3

Description: Every unique coordinate for where a taxa has been recorded in Antarctic, and the ACBR this coordinate falls into

File Name: Supplementary Data 4

Description: Species richness of raster grid cells across Antarctic, at 65km2 resolution. The points in the file represent the central point of each gridcell

File Name: Supplementary Data 5

Description: Common names of the chordates occurring in the dataset

File Name: Supplementary Data 6

Description: A table for all ASPAs, similar to Supplementary Table 2, but with a some additional fields, including the ID number, area, designation reasons and ACBR in which it occurs.

File Name: Supplementary Software 1

Description: SnapshotOfAntarcticBiodiversityCode.R contains code to run the full analysis and present all results given in the paper, in R, using the supplementary datasets (converted to .csv). Code was written and finalised using R version 3.4.1 (Single Candle) on a MacBookPro running MacOS Sierra 10.12.6.
