# Supplementary material for: A snapshot of biodiversity protection in Antarctica
Source: Nat Commun. 2019 Feb 26;10:946. doi: 10.1038/s41467-019-08915-6 (PMC6391489; doi:10.1038/s41467-019-08915-6)
Supplement: Supplementary file 4 — Supplementary Data 1 [file 41467_2019_8915_MOESM4_ESM.rtf]

This data and code is to analyse Antarctic biodiversity data in relation to its protection in ASPAs, as published in: Wauchope, H. S., Shaw, J., Terauds A. (2019) A snapshot of biodiversity protection in Antarctica. Nature Communications. Please direct any questions or comments to hannah.wauchope@uqconnect.edu.au

**********

First, there is the code file “SnapshotOfAntarcticBiodiversityCode.R”. This contains code to run the full analysis and present all results given in the paper, in R, using the following data. Code was written and finalised using R version 3.4.1 (Single Candle) on a MacBookPro running MacOS Sierra 10.12.6. (Full session info provided at bottom of document)

**********

The full dataset of geo-located species records is being prepared for publication as a data paper. In the meantime, three data files are presented, plus two additional files:

**********

1. AntarcticData.xslx: This file contains records of which species occur in each of the 16 Antarctic Conservation Biogeographic Regions (ACBRs,) and which species occur in each of the 72 Antarctic Specially Protected Areas (ASPAs). Used for Figure 1 and supplementary figures 1 and 2. Contains the following fields:

Taxon_ID: A unique identifier for each taxa
ASPA_No: The number of the ASPA the species occurs in (0 or NA for 'does not occur in an ASPA')
ACBR_ID: The number of the ACBR the species occurs in (0 or NA for 'does not occur in ACBR')
Protocol_designation: The primary protocol designation of the ASPA (or NA), see paper for details
BinaryProt: A binary value indicating a 1 if the species occurs in an ASPA or a 0 if not (For ease of analysis)
Biodiversity_designated: Is a 1 if the species occurs in an ASPA that is designated to protect biodiversity values
KINGDOM, PHYLUM, CLASS, ORDER, FAMILY, GENUS, SPECIES: Taxonomic information for the species
GENSPEC: Full species name (genus and species)

**********

2. AntarcticPoints.xslx: This file contains every unique coordinate for where a taxa has been recorded in Antarctic, and the ACBR this coordinate falls into. Used for Supplementary Figure 1d. Contains the following fields:

Latitude and Longitude: In standard WGS84 coordinate system
ACBR_ID: The ACBR_ID that the point falls in (0 if none)

**********

3. RichnessPoints.xslx: This file contains species richness of raster grid cells across Antarctic, at 65km2 resolution. The points in the file represent the central point of each gridcell (see Code for how this was created). Used in Supplementary Figure 3. Contains the following fields:

Lon_Stereo and Lat_Stereo: Coordinate of centre of raster gridcell in Polar Stereographic projection (CRS = “+proj=stere +lat_0=-90 +lat_ts=-71 +lon_0=0 +k=1 +x_0=0 +y_0=0 +datum=WGS84 +units=m +no_defs +ellps=WGS84 +towgs84=0,0,0") 

Lat_WGS and Lon_WGS: Coordinate of centre of raster gridcell in standard WGS84 coordinate system

Richness: Number of species occurring in that gridcell 

**********

4. ChordateCommonNames.xslx: This file contains the common names of the chordates occurring in the dataset, used for Figure 1d and Supplementary Figure 4. Contains the following fields:

Taxon_ID: A unique identifier for each taxa
CommonName: The common name of that taxa

**********

5. ASPA_Details.xslx: This file is the same as Supplementary Table 2, a table of data on each ASPA, with a few additional fields:

ASPA_No: The number of the ASPA 
Tot_Area: The total area of the ASPA
Protocol_designation: The primary protocol designation of the ASPA
Biodiversity_designated: Is 1 if the ASPA is designated to protect biodiversity values
Marine: Is a 1 if the ASPA is designated to protected a marine region
ACBR_ID: Gives the ACBR ID of the ACBR the ASPA occurs in, if any

Note ASPAs 114, 118 and 130 are de-designated

**********
Full R session info used to run this analysis, including all package versions:

R version 3.4.1 (2017-06-30)
Platform: x86_64-apple-darwin15.6.0 (64-bit)
Running under: macOS Sierra 10.12.6

attached base packages:
[1] stats     graphics  grDevices utils     datasets  methods   base     

other attached packages:
 [1] gstat_1.1-5       raster_2.5-8      data.table_1.10.4 rgeos_0.3-23      scales_0.5.0      stringr_1.2.0     extrafont_0.17    maptools_0.9-2    rgdal_1.2-8       sp_1.2-5          reshape2_1.4.2    reshape_0.8.7    
[13] ggplot2_2.2.1     dplyr_0.7.6      

loaded via a namespace (and not attached):
 [1] Rcpp_0.12.19     pillar_1.3.0     compiler_3.4.1   plyr_1.8.4       bindr_0.1.1      xts_0.10-0       tools_3.4.1      digest_0.6.12    tibble_1.4.2     gtable_0.2.0     lattice_0.20-35  pkgconfig_2.0.1 
[13] rlang_0.2.2      rstudioapi_0.7   bindrcpp_0.2.2   Rttf2pt1_1.3.6   grid_3.4.1       tidyselect_0.2.5 spacetime_1.2-1  glue_1.3.0       R6_2.2.2         foreign_0.8-69   purrr_0.2.5      extrafontdb_1.0 
[25] magrittr_1.5     intervals_0.15.1 assertthat_0.2.0 colorspace_1.3-2 labeling_0.3     stringi_1.1.5    lazyeval_0.2.0   munsell_0.4.3    FNN_1.1          crayon_1.3.4     zoo_1.8-0  
