# Supplementary material for: A snapshot of biodiversity protection in Antarctica
Source: Nat Commun. 2019 Feb 26;10:946. doi: 10.1038/s41467-019-08915-6 (PMC6391489; doi:10.1038/s41467-019-08915-6)
Supplement: Supplementary file 11 — Reporting Summary [file 41467_2019_8915_MOESM11_ESM.pdf]

nature research

Corresponding author(s): Aleks Terauds

Reporting Summary

Nature Research wishes to improve the reproducibility of the work that we publish. This form provides structure for consistency and transparency in reporting. For further information on Nature Research policies, see Authors & Referees and the Editorial Policy Checklist.

Statistical parameters

When statistical analyses are reported, confirm that the following items are present in the relevant location (e.g. figure legend, table legend, main text, or Methods section).

n/a	Confirmed	
☐	☒	The exact sample size (n) for each experimental group/condition, given as a discrete number and unit of measurement
☒	☐	An indication of whether measurements were taken from distinct samples or whether the same sample was measured repeatedly
☒	☐	The statistical test(s) used AND whether they are one- or two-sided<br>*Only common tests should be described solely by name; describe more complex techniques in the Methods section.*
☒	☐	A description of all covariates tested
☒	☐	A description of any assumptions or corrections, such as tests of normality and adjustment for multiple comparisons
☒	☐	A full description of the statistics including central tendency (e.g. means) or other basic estimates (e.g. regression coefficient) AND variation (e.g. standard deviation) or associated estimates of uncertainty (e.g. confidence intervals)
☒	☐	For null hypothesis testing, the test statistic (e.g. F, t, r) with confidence intervals, effect sizes, degrees of freedom and P value noted<br>*Give P values as exact values whenever suitable.*
☒	☐	For Bayesian analysis, information on the choice of priors and Markov chain Monte Carlo settings
☒	☐	For hierarchical and complex designs, identification of the appropriate level for tests and full reporting of outcomes
☒	☐	Estimates of effect sizes (e.g. Cohen's d, Pearson's r), indicating how they were calculated
☒	☐	Clearly defined error bars<br>*State explicitly what error bars represent (e.g. SD, SE, CI)*

Our web collection on statistics for biologists may be useful.

Software and code

Policy information about availability of computer code

Data collection	No software was used to collect the data
Data analysis	Data was processed and analyzed using readily available R packages for data frame manipulations (e.g.dlyr, reshape) and GIS (raster etc). All code used to generate the summary statistics and figures has been provided in the source data file.

For manuscripts utilizing custom algorithms or software that are central to the research but not yet described in published literature, software must be made available to editors/reviewers upon request. We strongly encourage code deposition in a community repository (e.g. GitHub). See the Nature Research guidelines for submitting code & software for further information.

Data

Policy information about availability of data

All manuscripts must include a data availability statement. This statement should provide the following information, where applicable:

- Accession codes, unique identifiers, or web links for publicly available datasets
- A list of figures that have associated raw data
- A description of any restrictions on data availability

A reporting summary for this Article us available as a Supplementary Information file.
The spatially explicit biodiversity data from the Antarctic Specially Protected Area Management Plans are publicly available from the Australian Antarctic Data

Centre (https://dx.doi.org:10.26179/5c1b10c534c19). The revised and updated Antarctic Specially Protected Area spatial layers (points and polygons) are also available from the Australian Antarctic Data Centre (https://dx.doi.org:10.26179/5c1b15eedaeb6). The SCAR Antarctic Biodiversity Database, which underpinned the comparative analysis, is currently being prepared for submission as a data paper, and as such is not publicly available at this time. Once published these data will also be publicly available from the Australian Antarctic Data Centre. In the interim please contact the corresponding author for access to this dataset.

Source data underlying Figure 1a-d, and Supplementary Figures 1-4 are provided as a Source Data file.

Field-specific reporting

Please select the best fit for your research. If you are not sure, read the appropriate sections before making your selection.

☐ Life sciences ☐ Behavioural & social sciences ☒ Ecological, evolutionary & environmental sciences

For a reference copy of the document with all sections, see nature.com/authors/policies/ReportingSummary-flat.pdf

Ecological, evolutionary & environmental sciences study design

All studies must disclose on these points even when the disclosure is negative.

Study description

In this study we undertook the first continent-wide assessment of terrestrial biodiversity protection in Antarctica. For this study we proceeded in three clearly defined stages:
1) Digitize and consolidate spatially explicit data on Antarctic biological life
2) Assess and georectify the polygons representing the Antarctic Specially Protected Areas (ASPAs)
3) Quantify and summarize the protection of Antarctic biodiversity on a continental and regional scale using species occurrence data within and outside of ASPAs

Research sample

We built upon the Antarctic biodiversity database compiled and used by Terauds et al. 2012 (Div & Dist), by searching for and adding spatially explicit records from the published literature. We found data in 178 sources, adding 7364 records representing 440 species to the original database used by Terauds et al. 2012. We then extracted all spatially explicit biodiversity data reported in the 73 existing ASPA management plans adding a further 2438 records to the database. Where possible we quantified the uncertainty around these spatial locations, and documented the original source of the record. The final biodiversity database holds 48,602 records, 2292 unique species and represents the most comprehensive consolidation of Antarctic terrestrial biodiversity information yet compiled.

Sampling strategy

We did not sub-sample the data and utilized the entire database for our analyses.

Data collection

Data was collected from a range of sources including herbaria records, spatially explicit data from publications and ASPA Management Plans

Timing and spatial scale

The entire database spanned the period 1902 - 2016 and covers all ice free areas of the Antarctic continent

Data exclusions

First, any species records with a spatial uncertainty of more than 10 km were removed, as were points that were shifted more than 10 km to snap to the rock outcrop shapefile. All marine and ice breeding species were removed, with the exception of emperor penguins (Aptenodytes forsteri) who were included on the basis of the comprehensive information on their breeding locations (eg see http://www.penguinmap.com/Species/) and the fact that several ASPAs were designated specifically to protect this species. Antarctic fur seals (Arctocephalus gazella) and southern elephant seals (Mirounga leonina) were also excluded as data for these species were not compiled as part of the Antarctic biodiversity database. Microbial taxa (primary from the Kingdoms Bacteria, Chromista and Protozoa) were also removed due to lack of taxonomically robust and comprehensive data.

Reproducibility

This was not an experiment per se, and therefore the question of reproducibility is not applicable here

Randomization

As we used the entire database, and as this was not an experiment, randomization was not applicable to our study

Blinding

As we used the entire database, blinding was not relevant to our study

Did the study involve field work? ☐ Yes ☒ No

Reporting for specific materials, systems and methods

Materials & experimental systems

n/a	Involved in the study
☒	☐ Unique biological materials
☒	☐ Antibodies
☒	☐ Eukaryotic cell lines
☒	☐ Palaeontology
☒	☐ Animals and other organisms
☒	☐ Human research participants

Methods

n/a	Involved in the study
☒	☐ ChIP-seq
☒	☐ Flow cytometry
☒	☐ MRI-based neuroimaging
